# Resilience as a Personality Trait and Stress Coping Styles: A Cross-Sectional Analysis of a Paramedic Student Cohort

**DOI:** 10.3390/jcm14061878

**Published:** 2025-03-11

**Authors:** Kazimiera Hebel, Sylwia Jałtuszewska, Aleksandra Steliga, Tomasz Kłosiewicz, Daniel Ślęzak, Sebastian Głowiński

**Affiliations:** 1 Institute of Health Sciences, Pomeranian University of Słupsk, 64 Bohaterów Westerplatte Street, 76-200 Słupsk, Poland; kazimiera.hebel@upsl.edu.pl (K.H.); sylwia.jaltuszewska@upsl.edu.pl (S.J.); aleksandra.steliga@upls.edu.pl (A.S.); sebastian.glowinski@tu.koszalin.pl (S.G.); 2 Department of Medical Rescue, Faculty of Health Sciences, Poznań University of Medical Sciences, 7 Rokietnicka Street, 60-608 Poznań, Poland; 3 Department of Medical Rescue, Institute of Emergency Medical Services, Medical University of Gdańsk, 80-210 Gdańsk, Poland; daniel.slezak@gumed.edu.pl; 4 Institute of Physical Culture and Health, State Higher School of Vocational Education in Koszalin, 1 Leśna Street, 75-582 Koszalin, Poland

**Keywords:** resilience, paramedic, life satisfaction, stress, coping styles

## Abstract

**Background**/**Objectives**: Resilience, defined as the ability to adapt and cope effectively with stress, plays a crucial role in preparing candidates for the paramedic profession. This study aimed to assess the resilience intensity as a personality trait and identify stress-coping styles among paramedic candidates. **Methods**: A cross-sectional cohort study was conducted across multiple Polish universities offering bachelor’s degree programs in emergency medical services. The study included 138 participants (56 females, 82 males, aged 18–51). Data collection involved demographic surveys and standardized tools, including the Polish version of the Resilience Scale (SPP-25), the Satisfaction with Life Scale (SWLS), and the Coping Inventory for Stressful Situations (CISS). **Results**: The study revealed a positive correlation between resilience and age (*p* = 0.002). Males exhibited significantly higher resilience compared to females (*p* = 0.0004). While both genders demonstrated average life satisfaction (SWLS), men scored significantly higher (*p* = 0.0082). Task-oriented coping strategies were predominant among all participants, but females scored higher in emotion-oriented coping (*p* = 0.0003). Resilience was positively correlated with task-oriented coping (0.4872) and negatively correlated with emotion-oriented coping (−0.4727). **Conclusions**: These findings emphasize the importance of resilience in paramedic training and selection, as it significantly influences stress management and professional performance in high-pressure situations.

## 1. Introduction

Students preparing to become paramedics are a distinct group, characterized by unique predispositions and motivations. Their decision to enter this field reflects a strong commitment to helping others and an interest in emergency medicine [1]. The paramedic study program is demanding, covering both theoretical and practical medical knowledge, including subjects such as anatomy, physiology, pharmacology, resuscitation, and first aid techniques.

Paramedic students must acquire the ability to act swiftly and effectively in critical situations, often under intense time pressure. This requires not only a solid theoretical foundation but also practical skills gained through numerous practical classes, simulation training, and internships in ambulance and emergency department. Paramedics must be mentally resilient, effective team players, and capable of making quick decisions in high-pressure situations. Many also maintain a high level of physical fitness, enabling them to work in demanding environments. Their training prepares them to become frontline professionals in life-saving interventions.

Resilience, defined as the ability to adapt and manage stress effectively, is crucial in preparing paramedic candidates. Resilience is associated with experiencing positive emotions, which allows individuals to view stressful situations as challenges and choose more adaptive coping strategies. Resilient individuals handle difficulties better, although they are not immune to stress. According to Wagnild et al., resilience enhances resistance to stress and supports the ability to maintain high-quality performance despite adversity [2].

Block et al. [3] described resilient individuals as warm, capable of forming close relationships, self-confident, productive, persistent, aware of their motivations, and consistent in pursuing goals. Other researchers emphasized additional traits, such as optimism, inner peace, high energy, curiosity about the world, and openness to new experiences [4,5,6,7]. Resilience incorporates cognitive aspects, like beliefs and expectations about reality, as well as emotional aspects, such as positive affect and emotional stability. Behaviorally, it manifests in seeking new experiences and employing various strategies to address problems [8].

Research on resilience as a personality trait shows it is linked to constructs such as emotional stability, openness to experience, optimism, sense of coherence, control, and self-efficacy. Resilience, when viewed as a self-regulation mechanism, is universal and protects against the negative effects of both severe trauma and everyday stress. The authors of the SPP-25 scale [9] demonstrated its value in measuring the personality predispositions of individuals exposed to stress, making it a useful tool for selecting candidates for professions like firefighting, policing, or paramedicine.

Findyartini et al. found that coping mechanisms, personality traits, and academic performance in medical students may be related to resilience, and that adaptive coping strategies are essential for enhancing resilience [10]. Research by Deldar and Yun [11,12] also highlighted that resilience helps manage stress and prevent burnout, which is particularly important in healthcare professions. Measuring resilience during the education of future paramedics and understanding the factors that influence it could be useful in assessing the suitability of candidates for this profession.

In Canada, Anderson and colleagues assessed the impact of an online training program on resilience among paramedic students, finding that participants demonstrated a significant improvement in resilience after completing the training [13]. Furthermore, Coyte et al. conducted an integrative review on resilience, post-traumatic growth, and psychological well-being among paramedicine clinicians, emphasizing the importance of psychological support programs in fostering resilience [14]. These international comparisons highlight the need to consider both individual and external factors in resilience-building strategies and point to potential improvements that could be implemented in paramedic education programs worldwide.

Burnout presents a significant challenge for paramedics, affecting their retention in the profession. Studies indicate that burnout prevalence ranges from 16% to 56% [15] with up to 60% of Polish paramedics reporting moderate to high levels of burnout [16]. Occupational and post-traumatic stress as well as emotional exhaustion contribute to alcohol and substance use and an increased risk of suicide among paramedics [17,18].

Therefore, it is crucial to develop strategies to identify and reduce the risk of burnout early in the recruitment and education process. In light of the importance of resilience, this study seeks to address the following questions:

1. Does resilience, as a personality trait, predispose individuals to effectively cope with stress?
2. What stress-coping styles are demonstrated by resilient individuals?
3. What motives influence students to pursue a career as a paramedic, and how do these motives relate to resilience, life satisfaction, and stress-coping styles?

## 2. Materials and Methods

### 2.1. Study Design and Setting

This cross-sectional cohort study was conducted between 1 January 2022 and 31 December 2023 at three Polish universities offering bachelor’s degree programs in paramedic science (Gdańsk, Poznań, Słupsk). First-year students were administered paper-based surveys. Initially, 156 questionnaires were returned, of which 18 were excluded due to incomplete completion of the Resilience Scale. Thus, the final study group comprised 138 students. Participants were informed of the study’s objectives, and anonymity was assured. Completion of the survey implied informed consent to participate.

### 2.2. Research Tools

Demographic data were collected using an author-designed survey. In addition, the following standardized tools were utilized:

1. Resilience Scale (SPP-25)

The Polish version of the Resilience Scale (SPP-25), developed by Ogińska-Bulik and Jurczyński [9], assesses resilience through 25 self-descriptive statements. Respondents rate each statement on a 5-point Likert scale, ranging from 0 (definitely no) to 4 (definitely yes). Higher scores indicate greater resilience. In addition to a total score, the scale assesses five factors: (1) perseverance and determination, (2) openness to new experiences and a sense of humor, (3) personal coping competencies and tolerance for negative emotions, (4) tolerance for failure and treating life as a challenge, and (5) an optimistic attitude and the ability to mobilize oneself in difficult situations. This scale has been shown to be a valid tool for assessing the suitability of individuals for high-stress professions such as firefighting, policing, and paramedicine.

2. Satisfaction with Life Scale (SWLS)

Jankowski’s Polish adaptation [19] of the Satisfaction with Life Scale (SWLS) [20] was used to measure overall life satisfaction. The SWLS consists of five statements rated on a 7-point Likert scale, where participants indicate how well each statement describes their life experience. The scores range from 5 to 35, with higher scores reflecting greater life satisfaction. Interpretation of results is based on ten scores, with the scale demonstrating a Cronbach’s alpha of 0.81, indicating good internal reliability.

3. Coping Inventory for Stressful Situations (CISS)

The Polish adaptation of the Coping Inventory for Stressful Situations (CISS) by Szczepaniak et al. [21], based on Endler and Parker’s original work [22], was employed to assess the frequency of various coping styles in stressful situations. The CISS evaluates three distinct coping styles:

○ Task-Oriented Style (TOS): High scores indicate a preference for problem-solving and cognitive effort aimed at altering the situation.
○ Emotion-Oriented Style (EOS): High scores suggest a focus on emotional responses, such as anger or guilt, and a tendency towards fantasizing or wishful thinking.
○ Avoidance-Oriented Style (AOS): High scores reflect avoidance behaviors, either through substitute activities (e.g., sleeping, watching TV) or seeking social contacts. The internal consistency (Cronbach’s alpha) of the TOS and EOS scales ranged from 0.82 to 0.88, while the AOS scale demonstrated alpha values between 0.74 and 0.78, indicating satisfactory reliability.

### 2.3. Ethical Considerations

This study was conducted in accordance with the principles outlined in the Declaration of Helsinki and adhered to the Strengthening the Reporting of Observational Studies in Epidemiology (STROBE) guidelines. All participants were informed of their rights. According to Polish law, the protocol of this study did not meet the criteria for a medical experiment, and the consent of the Bioethics Committee was not required (Decision of the Bioethics Committee of Poznań University of Medical Sciences No: KB63324).

### 2.4. Statistical Analysis

Statistical analyses were performed using Statistica 13.3 software. Quantitative variables were described using arithmetic means, standard deviations, medians, ranges (minimum and maximum), and 95% confidence intervals (CIs). Qualitative variables were presented as frequencies and percentages. The Grubbs’ test was initially applied to identify outliers. The normality of data distribution was assessed using the Shapiro–Wilk, Lilliefors, Kolmogorov–Smirnov, and Jarque–Bera tests. The Levene’s test was used to evaluate the homogeneity of variances. For comparison of two independent groups, the Student’s t-test was applied if variances were homogeneous; otherwise, the Mann–Whitney U-test was used. When comparing more than two groups without normal distribution, the Kruskal–Wallis test was employed, followed by post hoc testing if statistically significant differences were observed. For dependent variables, the Wilcoxon signed-rank test was applied where appropriate [23]. For qualitative data, chi-square, Pearson’s chi-square, and Fisher’s exact tests were utilized as needed. Graphical representations were used to illustrate relationships between variables and identify potential outliers. All statistical tests were two-tailed, and a significance level of *p* = 0.05 was applied. Significant ***p***-values were annotated on corresponding graphs.

## 3. Results

### 3.1. Study Group Characteristics

The study group consisted of 138 participants, of which 56 were females (40.6%) and 82 were males (59.4%). Participants’ ages ranged from 18 to 51 years, with a mean age of 22.2 years (SD = 6.1) and a median age of 20 years. A significant difference in age was observed between genders, with females being younger than males (*p* = 0.0018) (Table 1).

Table 2 presents the primary reasons participants chose the paramedic profession. The predominant motivation, cited by 89 respondents (64.5%), was the desire to save lives. Other factors, such as the public’s trust in the profession and opportunities for further education, played a less significant role in their decision to pursue this career.

### 3.2. Resiliency (SPP-25)

The study results indicated a significant positive correlation between resilience and the age of the respondents (*p* = 0.002). Table 3 presents the overall resilience scores as well as gender-specific results. Male participants exhibited significantly higher resilience levels compared to females (*p* = 0.0004). Notably, significant differences were observed across three factors of the resilience scale. Men demonstrated greater personal coping competencies and a higher tolerance for negative emotions than women (*p* = 0.0001). Additionally, men scored higher in their tolerance for failure and their perception of life as a challenge (*p* = 0.007). An optimistic attitude and the ability to mobilize oneself in difficult situations were also more prominent among men (*p* = 0.0001).

### 3.3. Life Satisfaction (SWLS) and the Styles of Coping with Stress (CISS)

Life satisfaction (SWLS) was at an average level for both genders; however, men obtained significantly higher results. Men showed higher life satisfaction (SWLS values) than women (*p* = 0.0082). Among the styles of coping with stress, the task-oriented style dominated in both groups. Women obtained higher values in the CISS EOS (emotion-oriented style) (*p* = 0.0003) (Table 4).

Table 5 presents descriptive statistics for the study group, focusing on statistically significant differences based on participants’ reasons for choosing to study paramedic science. Participants who selected this field as their dream profession scored significantly higher on the Satisfaction with Life Scale (SWLS) (*p* = 0.0384). Conversely, individuals whose primary motivation was not to save lives had higher scores on the emotion-oriented coping style (CISS EOS) (*p* = 0.0195).

Higher scores in the task-oriented coping style (CISS TOS) were observed among participants who cited the opportunity to work in various healthcare settings (*p* = 0.0175) and those expressing a desire to work abroad (*p* = 0.0029). These same individuals also demonstrated lower scores in the avoidance-oriented style of coping, specifically in the domain of engaging in substitute activities (*p* = 0.0210). Additionally, participants who indicated a willingness to work abroad scored higher on the resilience measurement scale (SPP-25) (*p* = 0.0061).

A higher tendency toward the searching for social contacts style (CISS SSC) was noted among participants who expressed a desire to work abroad (*p* = 0.0065) and those influenced by family members practicing in the medical field.

Table 6 summarizes the correlation analysis results, with statistically significant correlations highlighted. Age demonstrated a moderate positive correlation with the sense of coercion scale (r = 0.2636), while other correlations involving age were weak and statistically insignificant. Life satisfaction (SWLS) showed a strong positive correlation with resilience (SPP-25) (r = 0.5511) and a significant negative correlation with the emotion-oriented coping style (EOS) (r = −0.4476). Additionally, SWLS had a positive correlation with the searching for social contacts style (SSC) (r = 0.2569).

The correlation between resilience (SPP-25) and task-oriented coping (TOS) was moderate (r = 0.4872), suggesting that individuals with higher resilience are more likely to adopt problem-solving approaches under stress. The relationship between resilience and emotion-oriented coping (EOS) was also moderate but negative (r = −0.4727), indicating that more resilient individuals tend to rely less on emotion-focused coping strategies.

The avoidance-oriented coping style (AOS) showed a strong correlation with engaging in substitute activities (ESA) (r = 0.8436), highlighting a close association between these behaviors. Some correlations, while statistically significant, were weak in effect size. For example, the correlation between SWLS and CISS PKT (r = 0.2569) suggests a relationship, but one that is relatively small in magnitude and should be interpreted with caution. In contrast, moderate correlations (r = 0.3–0.6) indicate meaningful but not definitive relationships, while strong correlations (r > 0.6) suggest robust associations between variables.

## 4. Discussion

The results of this study provide valuable insights into the role of resilience as a personality trait and its association with stress-coping styles among paramedic candidates. Our findings highlight the multifaceted nature of resilience and its significant implications for the recruitment and training of future paramedics. Specifically, resilience was found to positively correlate with age, suggesting that older candidates, possibly due to accumulated life experiences, may possess greater resilience. This insight underscores the potential benefit of considering maturity during the recruitment process.

Gender differences were also notable, with men reporting higher resilience and life satisfaction. As Jachimowicz et al. [24] suggest, such traits may enhance coping mechanisms and activity in challenging situations—qualities that are essential in paramedicine. Understanding these demographic differences can guide tailored support and training programs to meet the varied needs of paramedic students.

Findyartini et al. [10] conducted a study among medical students to examine the relationship between resilience and factors such as personality traits, coping mechanisms, and academic performance. Their findings indicated that resilience is significantly related to these individual factors, with adaptive coping mechanisms playing a crucial role in enhancing resilience among medical students. This highlights the importance of personal factors in supporting resilience, which could be applied to ensure personalized learning and student support. Similarly, our study found a significant relationship between resilience and various coping styles.

Resilient individuals in our study demonstrated a preference for task-oriented coping strategies and seeking social contacts, both of which are adaptive and effective in managing stress. These strategies promote problem-solving and social support. Conversely, resilience was negatively correlated with the emotion-oriented coping style. This suggests that resilient individuals are less likely to engage in maladaptive coping behaviors, such as excessive emotional expression or rumination. Women in our study showed significantly higher scores in the emotion-oriented style (EOS), a coping approach characterized by focusing on emotions and engaging in wishful thinking or fantasizing [25].

Motivations behind choosing the paramedic profession also significantly influenced resilience and coping styles. Candidates driven by altruistic motives, such as a desire to save lives, reported higher life satisfaction and demonstrated more adaptive, task-oriented coping strategies. In contrast, candidates motivated by non-altruistic factors were more likely to rely on emotion-oriented coping strategies. This strategy may be less effective in the high-stress environment of paramedic work (as observed in gender differences: men vs. women). Additionally, candidates who aspired to work abroad exhibited higher resilience and task-oriented coping, indicating a proactive and goal-oriented mindset, essential traits for thriving in challenging environments. Those influenced by family members in the medical profession demonstrated a preference for social-oriented coping mechanisms, underscoring the role of social support networks in fostering resilience. The results obtained are consistent with the definition of a resilient person given by different authors [3,4,5,6,7], as warm, capable of forming close relationships, self-confident, aware of their motivations, and open to new experiences. Coyte B. et al. [14] found that higher resilience scores were associated with some relatively stable factors, such as a sense of coherence and personality traits “*bold*, *diligent*, *colourful* and *imaginative*” [23]. Positive strategies are used by individuals with these characteristics to manage stress, as confirmed in our research. This finding correlates with Weber et al. [26], who also identified altruistic motives as the main motivating factors for pursuing studies in the field of emergency services.

A qualitative study by Wahab et al. analyzed the experiences of paramedic students from Saudi Arabia and the United Kingdom, identifying stress-inducing factors affecting their well-being. The findings indicate cultural differences in the perception of stress and coping mechanisms, which directly influence the level of psychological resilience among students in these countries [27].

These findings have practical implications for paramedic recruitment and training. Firstly, integrating resilience assessments, such as the SPP-25 scale, into the candidate selection process could help identify those who are better prepared to cope with the challenges of the profession [28]. Previous studies emphasize the need to develop resilience among healthcare professionals, including nurses, to increase work efficiency and prevent burnout [11,12]. Tools like the SPP-25 scale offer a reliable measurement of resilience and can serve as a useful selection criterion alongside other competency. Second, training programs should focus on developing adaptive coping strategies through resilience-building exercises, stress management workshops, and simulations of real-life emergency scenarios. Additionally, support systems, including mentoring programs and peer support groups, can enhance resilience by fostering a sense of community and shared experience. The need to introduce programs aimed at strengthening resilience is indicated by many researchers, including. Coyte B et al. [14] and Clompus SR et al. [29]. These programs should focus on the coping strategies identified in this study. Active coping strategies should be strengthened during training, while dysfunctional coping styles should be addressed to promote behavioral change. Training is recommended not only for students but also for paramedics as part of continuous professional development. Given the importance of resilience, the study will be repeated next year with the same group of students to further explore these findings.

### Limitations

The authors are aware of several limitations that should be acknowledged. The cross-sectional design captures a snapshot in time, and longitudinal studies are needed to assess how resilience and coping styles evolve throughout the paramedic training program and into professional practice. Our research will continue. The aim of further analysis will be to assess the change in resilience after completing three-year studies. We also expect to identify weaknesses in education to enhance the effect of psychological preparation for self-employment. Furthermore, the study does not account for socio-economic status or family background, which may significantly influence resilience. Prior research suggests that financial stability and strong social support systems can enhance an individual’s ability to cope with stress, yet these factors were not explicitly analyzed in this study. Future research should incorporate these variables to gain a more comprehensive understanding of resilience determinants in paramedic students. Additionally, the use of self-reported measures may introduce a response bias, as participants may not always accurately reflect their coping behaviors or resilience levels. Future studies could incorporate objective assessments of resilience and coping strategies to reduce this potential bias. Moreover, in our study, the sample size was determined based on data availability and feasibility rather than a formal power analysis. However, we recognize that conducting a power analysis would strengthen the methodological rigor of our research. In future studies, we plan to incorporate a priori power calculations to ensure that the sample size is sufficiently powered to detect meaningful differences.

Further research could also examine the effectiveness of resilience-building interventions on stress management and job performance among paramedic students [30,31]. Additionally, exploring other personality traits such as emotional intelligence and empathy, alongside resilience, would provide a more comprehensive understanding of the traits contributing to effective paramedic practice.

## 5. Conclusions

The results of this study demonstrate that individuals with higher resilience tend to prefer task-oriented stress-coping styles, a trait that aligns well with the demands of the paramedic profession. Our findings underscore the importance of incorporating resilience into the recruitment and training processes for paramedics. By fostering resilience and encouraging the development of adaptive coping strategies, training programs can better prepare future paramedics to manage the high-pressure situations they will encounter, ultimately enhancing both their well-being and job performance.

## Figures and Tables

**Table 1:** Characteristics of the study group.

Variable	Parameters	Total (n = 138)	Male (n = 82)	Female (n = 56)	*p*-Value	d-Cohen
Age [years]	Mean (SD) [95% CI] Me Range	22.2 (6.1) [21.2; 23.3] 20 18–51	23.6 (7.1) [22.0; 25.1] 20 18–51	20.3 (3.3) [19.5; 21.2] 19 18–41	0.0018 *	0.5961

* Student’s *t*-test.

**Table 2:** Reasons for choosing the profession of a paramedic.

Purpose		Purpose	
The desire to save human life	Yes—89 (64.5%) No—49 (35.5%)	Possibility of further education	Yes—10 (7.2%) No—128 (92.8%)
The profession of my dreams	Yes—22 (15.9%) No—116 (84.1%)	Good financial conditions	Yes—20 (14.5%) No—118 (85.5%)
Possibility to work in health units	Yes—28 (20.3%) No—110 (79.7%)	Willingness to go to work abroad due to better working conditions and pay	Yes—12 (8.7%) No—126 (91.3%)
Possibility to work in military units and fire brigades	Yes—26 (18.8%) No—112 (81.2%)	Under the encouragement of people from the immediate family	Yes—16 (11.6%) No—122 (88.4%)
A profession of public trust	Yes—10 (7.2%) No—128 (92.8%)	Other reasons	Yes—17 (12.3%) No—121 (87.7%)

**Table 3:** The resilience of the study group, Mean (SD), [95% CI], Me, Range.

Variable		Total (n = 138)	Female (n = 56)	Male (n = 82)	t *	*p*-Value
Resilience—the overall result	Mean (SD) [95% CI] Me Range	74.63 (10.5) [72.9;76;4] 76 46–98	70.93 (9.62) [68.4;73.5] 72 47–91	77.21 (10.38) [74.9;79.5] 77 46–98	3.614	0.0004
1. Perseverance and determination in action		14.41 (3.05) [13.9;14.9] 15 7–20	14.10 (3.07) [13.3;14.9] 14 7–20	14.62 (3.04) [13.9;15.3] 15 7–20	0.981	0.328
2. Openness to new experiences and a sense of humor		16.37 (2.24) [15.9;16.7] 17 7–20	16.05 (2.02) [15.5;16.6] 16 10–20	16.60 (2.37) [16.1;17.1] 15 7–20	1.414	0.159
3. Personal coping competencies and tolerance for negative emotions		14.97 (2.73) [14.5;15.4] 15 6–20	13.81 (2.50) [13.1;14.5] 14 8–19	15.78 (2.61) [15.2;16.4] 16 6–20	4.462	0.0001
4. Tolerance for failure and treating life as a challenge		15.40 (2.52) [14.9;15.8] 16 8–20	14.72 (2.53) [14.1;15.4] 15 8–20	15.88 (2.43) [15.2;16.4] 16 8–20	2.723	0.007
5. Optimistic attitude toward life and the ability to mobilize oneself in difficult situations		14.47 (3.03) [12.9;13.9] 14 6–20	12.25 (2.84) [11.5;12.9] 13 6–17	14.33 (2.87) [13.7;14.9] 15 6–20	4.228	0.0001

* Student’s *t*-test.

**Table 4:** Life satisfaction and the styles of coping with stress (Mean (SD), [95% CI], Me, Range).

Variable	Total (n = 138)	Male (n = 82)	Female (n = 56)	*p*-Value	d-Cohen
Life satisfaction SWLS	23.1 (5.14) [22.2; 24.0] 23 5–35	24.0 (5.1) [22.9; 25.1] 24 5–35	21.8 (4.9) [20.5; 23.1] 22 11–32	0.0082 *	0.4399
CISS TOS	60.8 (8.1) [59.5; 62.2] 61 37–80	61.5 (8.3) [59.7;63.4] 62.5 42–78	59.9 (7.7) [57.8;61.9] 60 37–80	0.2177 **	0.200
CISS EOS (emotion-oriented style)	40.9 (11.6) [38.9; 42.8] 40 18–69	37.9 (10.7) [35.5; 40.2] 35 18–65	45.3 (11.6) [42.2; 48.4] 44.5 21–69	0.0003 *	0.6631
CISS AOS	42.5 (10.1) [40.8; 44.3] 42 22–68	41.0 (8.9) [39.8;43;8] 42 22–63	42.8 (12.8) [39.4;46.2] 43 0–68	0.5847 *	0.1659
CISS ESA	17.9 (6.0) [16.9; 18.9] 17 8–33	17.03 (5.5) [15.8;18.2] 17 8–32	18.8 (6.9) [16.9;20.6] 18 0–33	0.1225 *	0.2855
CISS SSC	16.2 (4.7) [15.4; 17.0] 16.5 5–25	16.5 (3.8) [15.7;17.4] 16.5 6–25	15.4 (5.9) [13.8;17.01] 16 0–25	0.382 *	0.2268

Data are presented as: * Mann–Whitney U-test, ** Student’s *t*-test; TOS—task-oriented style; EOS—emotion-oriented style; AOS—avoidance-oriented style; ESA—engaging in substitute activities; SSC—searching for social contacts.

**Table 5:** Study group divided by reasons for choosing to study paramedic science.

	The Profession of My Dreams		*p*-Value	d-Cohen Test Power
	Yes (n = 22)	No (n = 116)		
SWLS	25.4 (4.7) [23.3; 27.5] 26 16–35	22.7 (5.1) [21.7; 23.6] 23 5–34	0.0384 *	0.5506 0.6181
	**The Desire to Save Human Life**		***p*-Value**	**d-Cohen** **Test Power**
	**Yes (n = 89)**	**No (n = 49)**		
CISS EOS	38.0 (12.8) [32.3; 43.6] 34.5 18–63	41.4 (11.4) [39.3; 43.5] 41 18–69	0.0195 *	0.2805 0.3169
	**Possibility to Work in Health Units**		***p*-Value**	**d-Cohen** **Test Power**
	**Yes (n = 26)**	**No (n = 112)**		
CISS TOS	62.3 (8.1) [59.0; 65.6] 61 37–80	60.5 (8.1) [59.0; 62.0] 61 37–80	0.0175 **	0.2222 0.1735
CISS ESA	17.2 (5.6) [15.0; 19.5] 17 8–27	18.0 (6.1) [16.9; 19.5] 17 8–33	0.0210 *	0.1366 0.0919
	**Willingness to Work Abroad**		***p*-Value**	**d-Cohen** **Test Power**
	**Yes (n = 12)**	**No (n = 126)**		
SPP-25	82.0 (7.6) [77.2; 86.8] 83 67–90	73.9 (10.5) [72.0; 75.7] 75 46–98	0.0061 *	0.8838 0.7175
CISS TOS	67.4 (5.9) [63.6; 71.2] 67 57–76	60.2 (8.0) [58.8; 61.6] 61 37–80	0.0029 ***	1.0243 0.8409
CISS SSC	19.8 (3.6) [17.5; 22.1] 19.5 15–25	15.9 (4.6) [15.0; 16.7] 16 5–25	0.0065 *	0.9442 0.7957
	**Influenced by Family Encouragement**		***p*-Value**	**d-Cohen** **Test Power**
	**Yes (n = 16)**	**No (n = 122)**		
CISS SSC	18.6 (4.2) [16.4; 20.9] 19 9–25	15.9 (4.6) [15.1; 16.7] 16 5–25	0.0244 *	0.6130 0.5917

Data are presented as: Mean (SD), [95% CI], Me, Range; * Mann–Whitney U-test, ** test with independent variance estimation (Welch), *** Student’s *t*-test; TOS—task-oriented style; EOS—emotion-oriented style; ESA—engaging in substitute activities; SSC—searching for social contacts.

**Table 6:** Correlations between age and resilience (SPP-25), life satisfaction (SWLS), styles of coping with stress (CISS).

	The Bolded Correlation Coefficients Are Significant with *p* < 0.05							
Variable	Age [years]	SWLS	SPP-25	CISS SSZ	CISS SSE	CISS SSU	CISS ACZ	CISS PKT
Age [years]	1.0000	0.1259	0.2636	0.1514	−0.1393	0.0834	−0.0083	0.1477
SWLS	0.1259	1.0000	0.5511	0.1282	−0.4476	0.0082	−0.1370	0.2569
SPP-25	0.2636	0.5511	1.0000	0.4872	−0.4727	0.0575	−0.1336	0.3088
CISS TOS	0.1514	0.1281	0.4872	1.0000	−0.1098	0.0593	−0.0606	0.2246
CISS EOS	−0.1393	−0.4476	−0.4727	−0.1098	1.0000	0.2711	0.3865	−0.0870
CISS AOS	0.0834	0.0082	0.0575	0.0593	0.2711	1.0000	0.8436	0.7176
CISS ESA	−0.0083	−0.1370	−0.1336	−0.0606	0.3865	0.8436	1.0000	0.2890
CISS SSC	0.1477	0.2569	0.3088	0.2246	−0.0870	0.7176	0.2890	1.0000

TOS—task-oriented style; EOS—emotion-oriented style; AOS—avoidance-oriented style; ESA—engaging in substitute activities; SSC—searching for social contacts.

## Data Availability

Data can be obtained from kazimiera.hebel@upsl.edu.pl.

## Abbreviations

The following abbreviations are used in this manuscript:

AOS	Avoidance-Oriented Style
CISS	Coping Inventory for Stressful Situations
EOS	Emotion-Oriented Style
SPP-25	Resilience Scale
SWLS	Satisfaction with Life Scale
SSC	Searching for Social Contacts Style
TOS	Task-Oriented Style
